# Periostin plays a critical role in the cell cycle in lung fibroblasts

**DOI:** 10.1186/s12931-020-1299-0

**Published:** 2020-01-30

**Authors:** Tomohito Yoshihara, Yasuhiro Nanri, Satoshi Nunomura, Yukie Yamaguchi, Carol Feghali-Bostwick, Keiichi Ajito, Shoichi Murakami, Masaaki Mawatari, Kenji Izuhara

**Affiliations:** 1 Division of Medical Biochemistry, Department of Biomolecular Sciences, Saga Medical School, 5-1-1 Nabeshima, Saga, 849-8501 Japan; 2 Department of Environmental Immuno-Dermatology, Yokohama City University Graduate School of Medicine, Yokohama, 236-0004 Japan; 3 Division of Rheumatology and Immunology, Department of Medicine, Medical University of South Carolina, Charleston, SC 29425 USA; 4 Pharmaceutical Research Center, Meiji Seika Pharma Co. Ltd., Yokohama, 222-8567 Japan; 5 Department of Orthopaedic Surgery, Saga Medical School, Saga, 849-8501 Japan

**Keywords:** Idiopathic pulmonary fibrosis, Periostin, Fibroblast, Proliferation, Cell cycle, Integrin

## Abstract

**Background:**

Idiopathic pulmonary fibrosis (IPF) is a devastating disease with a median survival of only three to 5 years. Fibroblast proliferation is a hallmark of IPF as is secretion of extracellular matrix proteins from fibroblasts. However, it is still uncertain how IPF fibroblasts acquire the ability to progressively proliferate. Periostin is a matricellular protein highly expressed in the lung tissues of IPF patients, playing a critical role in the pathogenesis of pulmonary fibrosis. However, it remains undetermined whether periostin affects lung fibroblast proliferation.

**Methods:**

In this study, we first aimed at identifying periostin-dependently expressed genes in lung fibroblasts using DNA microarrays. We then examined whether expression of cyclins and CDKs controlling cell cycle progression occur in a periostin-dependent manner. We next examined whether downregulation of cell proliferation-promoting genes by knockdown of periostin or integrin, a periostin receptor, using siRNA, is reflected in the cell proliferation of lung fibroblasts. We then looked at whether lung fibroblasts derived from IPF patients also require periostin for maximum proliferation. We finally investigated whether CP4715, a potent inhibitor against integrin α_V_β_3_ (a periostin receptor), which we have recently found blocks TGF-β signaling, followed by reduced BLM-induced pulmonary fibrosis in mice, can block proliferation of lung fibroblasts derived from IPF patients.

**Results:**

Many cell-cycle–related genes are involved in the upregulated or downregulated genes by periostin knockdown. We confirmed that in lung fibroblasts, periostin silencing downregulates expression of several cell-cycle–related molecules, including the cyclin, CDK, and, E2F families, as well as transcription factors such as B-MYB and FOXM1. Periostin or integrin silencing slowed proliferation of lung fibroblasts and periostin silencing increased the distribution of the G0/G1 phase, whereas the distribution of the G2/M phase was decreased. Lung fibroblasts derived from IPF patients also required periostin for maximum proliferation. Moreover, CP4715 downregulated proliferation along with expression of cell-cycle–related genes in IPF lung fibroblasts as well as in normal lung fibroblasts.

**Conclusions:**

Periostin plays a critical role in the proliferation of lung fibroblasts and the present results provide us a solid basis for considering inhibitors of the periostin/integrin α_V_β_3_ interaction for the treatment of IPF patients.

## Background

Idiopathic pulmonary fibrosis (IPF) is a characteristically progressive chronic lung disease, with irreversible lung scarring and the histological features of interstitial fibrosis of lung tissues, and unknown etiology [1]. IPF is devastating; the median survival of IPF patients is 3 to 5 years. Aberrant activation of the lung epithelium triggers pulmonary fibrosis, producing mediators of fibroblast migration, proliferation, and differentiation into active myofibroblasts. The fibroblasts and myofibroblasts then secrete exaggerated amounts of extracellular matrix (ECM) proteins, which then remodel the lung architecture.

Fibroblast proliferation is a hallmark of IPF, as is the secretion of ECM proteins from fibroblasts [2]. It is still uncertain whether, in the lung tissues of IPF patients, the progressive proliferation of fibroblasts is programmed in fibroblasts themselves or is in some way influenced by the extracellular milieu. Several trials have been performed to compare gene profiles in fibroblasts derived from IPF patients and normal donors [3–5], showing that gene expression patterns differ in these two types of fibroblasts. Expression of fibrosis-related genes (*IGFBP3*, *IGFBP7*, *LOX*, and *POSTN*) chemokines (*CCL2*, *CCL8*, and *CCL2*6), and growth factors such as *FGF7* are upregulated in IPF fibroblasts. Several proliferation-related genes such as *WNT5A* and *RGCC* are sporadically observed in the gene profiles of upregulated genes in IPF fibroblasts. However, we are far from understanding how IPF fibroblasts acquire the ability to progressively proliferate.

Periostin encoded by the *POSTN* gene is a matricellular protein of 93.3 kDa in size belonging to the fasciclin family and is involved in the pathogenesis of various inflammatory and fibrotic diseases by accelerating inflammation or fibrosis [6, 7]. We and others have demonstrated that periostin is highly expressed in the lung tissue of IPF patients [8–12]. It is of note that expression of periostin is significant in fibroblastic foci in which fibrosis is active and that upon stimulation by either IL-4 or IL-13 periostin can be detected in the supernatant of lung fibroblasts, but not of airway epithelial cells [8–10, 13], suggesting that fibroblasts are main sources of periostin in lung. Moreover, we and another group have shown that a genetic deficiency of periostin or the administration of neutralizing antibodies (Abs) against periostin protected mice from bleomycin (BLM)-induced pulmonary fibrosis [10, 14], suggesting the significance of periostin in generating pulmonary fibrosis. Periostin acts by binding several integrin molecules—α_V_β_1_, α_V_β_3_, α_V_β_5_, α_6_β_4_, and α_M_β_2_—on cell surfaces [7]. We have previously shown that periostin derived from fibroblasts acts on fibroblasts by co-operating with inflammatory cytokines such as TNF-α activating NF-κB, followed by inducing pro-inflammatory cytokines or chemokines [14]. This is one underlying mechanism by which periostin causes pulmonary fibrosis. Moreover, we have recently found that cross-talk between TGF-β, a critical mediator for pulmonary fibrosis, and periostin via α_V_β_3_ integrin is important for generating pulmonary fibrosis [15]. However, it has remained undetermined whether or how periostin affects the proliferation of fibroblasts.

In this study, we first aimed to identify periostin-dependently expressed genes in lung fibroblasts, comparing the gene profile in periostin-silenced fibroblasts and found that many cell-cycle–related genes are involved in this profile. Accordingly, periostin- or integrin α_V_β_3_-silenced fibroblasts showed slower proliferation. Lung fibroblasts derived from IPF patients also required periostin for maximum proliferation. Moreover, an inhibitor of integrin α_V_β_3_, a periostin receptor, downregulated proliferation along with expression of cell-cycle–related genes in IPF lung fibroblasts as well as in normal lung fibroblasts. These results offer the first formal proof that periostin plays a critical role in the proliferation of lung fibroblasts.

## Methods

### Cell culture

MRC-5 cells (Riken BioResource Center, Tsukuba, Japan) were maintained as previously described [16]. NHLFs (normal human lung fibroblasts) were purchased from Lonza (Basel, Switzerland). RNA extracts were applied to quantitative reverse transcription PCR (qRT-PCR). Five clones of lung fibroblasts were cultured from the explanted lungs of IPF patients undergoing lung transplantation and nine clones of lung fibroblasts were also cultured from normal donor lungs that were not used for transplantation [3, 17].

### Knockdown of mRNA by siRNA

siRNA oligonucleotides were purchased from Dharmacon/GE Healthcare (Lafayette, CO, USA). Cells were transfected with ON-TARGET plus siRNA for *POSTN*, *ITGAV*, *ITGB3*, or control at the indicated concentrations and for the indicated times in the presence of RNAiMAX reagent (Thermo Fisher Scientific, Rockford, IL, USA).

### DNA microarray analysis

MRC-5 cells were transfected with 10 nM periostin siRNA for 48 h. Total RNA with an RNA integrity number greater than 9.2 was applied to Agilent Expression Array (SurePrint G3 Human GE8x60K v2 Microarray, Takara Bio, Shiga, Japan). The calculated relative signal intensity values were presented on a heat map and subjected to MultiExperiment Viewer (MeV) v4.9 software (Dana-Farber Cancer Institute, Boston, MA, USA). For gene ontology analysis, the Database for Annotation, Visualization, and Integrated Discovery (DAVID) tool (National Cancer Institute, Frederick, MD, USA) was used. This database includes the Gene Ontology Database (http://geneontology.org/, GSE132917).

### Cell proliferation assay

After incubation in serum-free medium for 24 h, cells were treated with control or with periostin siRNA. Cell proliferation was evaluated using a Cell Counting kit-8 (Dojindo, Kumamoto, Japan) or BrdU Cell Proliferation ELISA Kit (Abcam, Cambridge, UK).

### Flow cytometry

To analyze cell death, the cells were treated with 50 μg/mL cycloheximide (Wako, Osaka, Japan) or 50 ng/mL TNF-α (PeproTech, Rocky Hill, NJ, USA) or either control siRNA or periostin siRNA for the indicated times. After harvested cells were labeled with annexin V FITC (Apoptosis Detection Kit I, BD Biosciences, Tokyo, Japan) and propidium iodide (PI, Sigma-Aldrich, San Diego, CA, USA), the cells were subjected to flow cytometry analysis using FACSCalibur (BD Biosciences).

For cell cycle analysis, MRC-5 cells and NHLFs were treated with control siRNA or periostin siRNA and then were fixed in ice-cold 70% ethanol for 1 h. After washing, cells were incubated in PI staining buffer (50 μg/mL PI, 0.1 mg/mL RNase A and 0.5% Triton X-100) for 30 min at 37 °C in the dark. After staining with PI (Sigma-Aldrich), the DNA content was analyzed by flow cytometry.

### qRT-PCR

qRT-PCR was performed as previously described [15]. Primers for qRT-PCR are described in Additional file 2: Table S1.

### ELISA

ELISA for human periostin was performed using two rat anti-human periostin mAbs, SS18A and SS17B (Shino-test, Tokyo, Japan), as previously reported [15.

### Adhesion assay

MRC-5 cells were transfected with 10 nM periostin siRNA for 72 h. Detached cells in the culture medium were collected to the tube and centrifuged. The cells were resuspended in the culture medium and counted the number of cells.

### Recombinant human periostin protein

Recombinant human periostin protein was purchased from R&D systems (Minneapolis, MN, USA). Ten μg/mL of recombinant human periostin protein was added for the cell proliferation assay.

### Transient transfection

Overexpression of periostin was performed as previously described [15].

### α_V_β_3_ inhibitor

CP4715, an α_V_β_3_ integrin inhibitor, was prepared as previously described [18–21]. In some experiments, MRC-5 cells and IPF lung fibroblasts were treated with 1 μM CP4715 dissolved in DMSO for 24 h.

### Statistical analysis

Data are presented as mean ± SD. Statistical analyses were performed using the Prism 5.0 software (GraphPad Software, La Jolla, CA, USA). Significance was assessed using an unpaired or paired Student’s *t*-test. Values of *P* < 0.05 were considered statistically significant.

## Results

### Comparison of the gene profiles affected by periostin silencing in lung fibroblasts

**Fig. 1:**
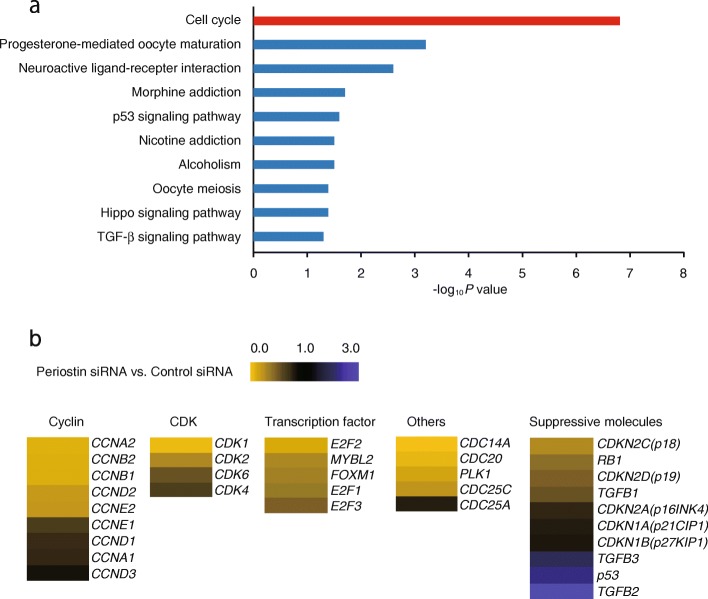
Comparison of the gene profiles affected by periostin silencing in lung fibroblasts. **a** MRC-5 cells with control or *POSTN*-specific siRNA (KD) were subjected to DNA microarray analysis. Genes downregulated by less than one third or genes upregulated by more than three-fold by knockdown of periostin were applied to DAVID analysis. Highly ranked GO terms are depicted. **b** Heat maps depict the genes in the GO term, cell cycle, with control siRNA versus periostin siRNA

To comprehensively identify genes expressed dependently on periostin in lung fibroblasts, we compared the gene expression profiles with or without knockdown of periostin in lung fibroblasts (MRC-5 cells). A total of 58,717 probes were tested using DNA microarrays; 1558 genes were downregulated to less than one third and 2097 genes were upregulated by more than three-fold, respectively, by knockdown of periostin (Additional file 3: Table S2). When we applied these genes to DAVID analysis to enrich biological functions in this group, a GO TERM, “cell cycle”, was ranked as the highest position (Fig. 1a). Downregulated genes included cell cycle-progression genes―cyclins, cyclin-dependent kinases (CDKs), cell division cycle (CDC) molecules, and several transcriptional factors―whereas upregulated genes included the cell cycle-suppressing genes p53 and TGF-β–relating genes (Fig. 1b). These results suggest that periostin plays a role in progressing the cell cycle in lung fibroblasts.

### Effect of periostin silencing on expression of cell cycle-related genes in lung fibroblasts

**Fig. 2:**
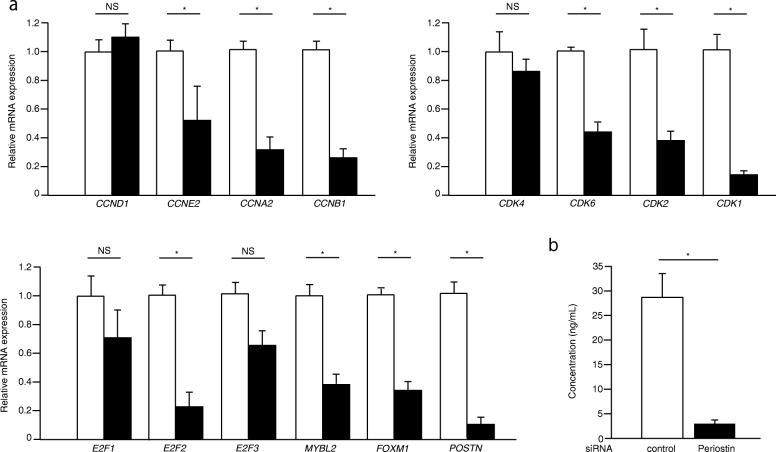
Effect of periostin silencing on expression of cell cycle-related genes in lung fibroblasts. MRC-5 cells were treated with 10 nM control (open box) or periostin (closed box) siRNA. **a** qRT-PCR for the indicated important cell cycle progression genes were performed in periostin-silenced MRC-5 cells after 48 h. The values were adjusted by *GAPDH* expression, and the fold changes are shown. **b** Periostin protein in the supernatant after 72 h. Values are mean ± SD of three independent experiments. *P < 0.05, NS: not significant

Cell cycle progression is strictly controlled by the complex of cyclins and CDKs; cyclin D/CDK 4 or 6 promotes progression into the G1 phase, cyclin E/CDK2 elicits G1/S transition, cyclin A/CDK 2 ensures progression in S and G2, and cyclin B/CDK1 brings about progression into the M phase [22, 23]. Moreover, it is known that synthesis of most cell cycle proteins is regulated at the transcriptional level [24]. We first examined whether expression of cyclins and CDKs controlling cell cycle progression occur in a periostin-dependent manner. In this experiment, we throughout analyzed mRNA expression of the indicated molecules. In MRC-5 cells, periostin knockdown downregulated expression of cyclins E2, A2, and B1, but not of cyclin D1; and CDKs 1, 2, and 6, but not 4 (Fig. 2a). Several members of the E2F family (E2F1–3) are known to promote the expression of G1 and S phase genes, whereas B-MYB and FOXM1 promote the expression of G2 and M phase genes [24–26]. Knockdown of periostin downregulated expression of E2F2 and of both B-MYB and FOXM (Fig. 2a). Expression of E2F1 and E2F3 tended to decrease, although not to a point of statistical significance. We confirmed decrease of periostin protein by the knockdown of periostin (Fig. 2b). However, we could not observe upregulation of the indicated genes by overexpression of periostin in MRC-5 cells (Additional file 1: Figure S1). These results suggest that expression of periostin is critical for broad expression of cell-cycle–promoting genes.

### Periostin or integrin silencing slows cell proliferation in lung fibroblasts

**Fig. 3:**
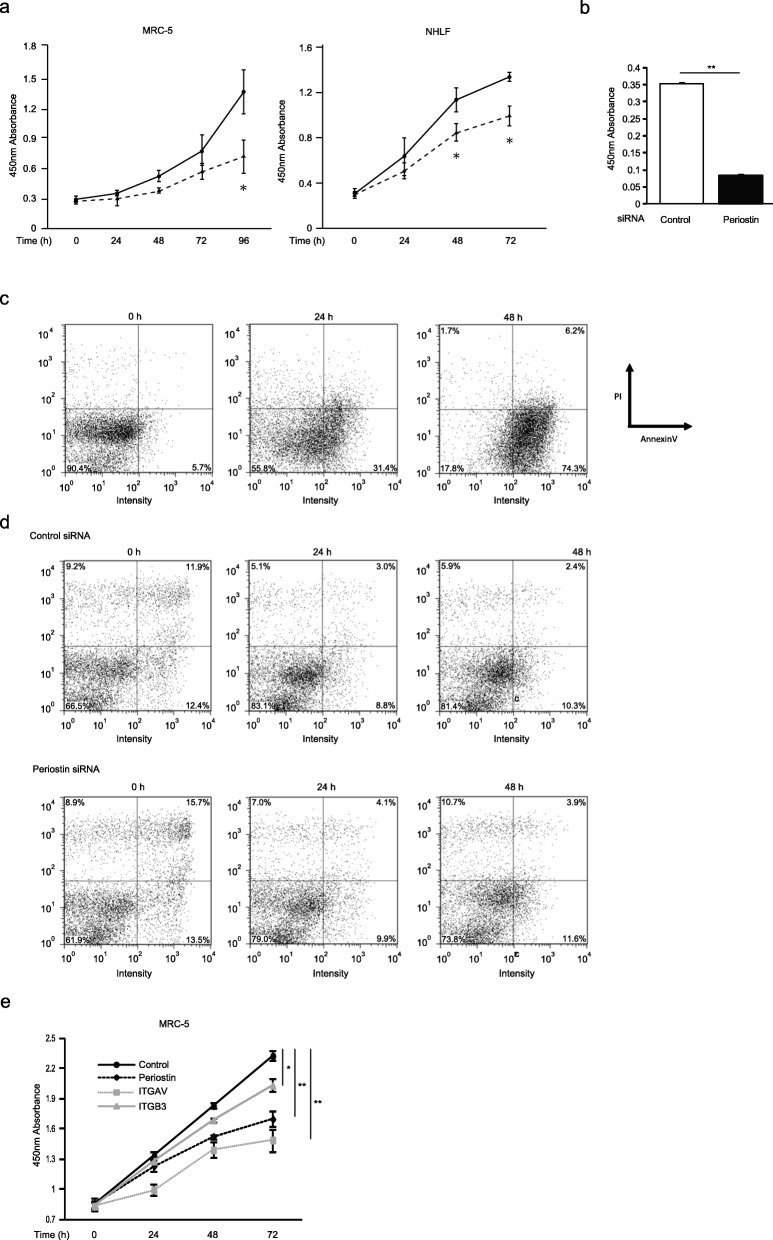
Periostin silencing slows cell proliferation in lung fibroblasts. **a**, **b**, **e** The growth curves of MRC-5 cells or NHLF. **a** The cells with (dashed line) or without (solid line) treatment of periostin knockdown were plated at a density of 1.0 × 10^4^ cells/well in 96-well plates. **b** The cells were treated with control siRNA or periostin siRNA for 48 h and pulsed with BrdU. After 12 h, the incorporation of BrdU was counted. Values are mean ± SD of three independent experiments. **e** The cells treated with siRNA for control (black solid line), periostin (black dashed line), α_V_ integrin (gray dashed line), or β_3_ integrin (gray solid line) were plated at a density of 1.0 × 10^4^ cells/well in 96-well plates. The cell numbers were evaluated at the indicated times. Values are mean ± SD of three independent experiments. *P < 0.05, **P < 0.01. **c**, **d** Flow cytometric analysis of annexin V (horizontal) and PI (vertical) labeling is depicted. The proportions of each fraction have been inserted. The same experiments were performed twice. MRC-5 cells were treated with 50 μg/mL cycloheximide and 50 ng/mL TNF-α (**c**) or siRNA for periostin (**d**) for the indicated times

To examine whether downregulation of cell proliferation-promoting genes by knockdown of periostin is reflected in the cell proliferation of lung fibroblasts, we assessed the effect of periostin silencing on the proliferation of MRC-5 cells and NHLFs. The number of cells treated with siRNA for periostin decreased significantly compared to the control cells at 96 h after the start of incubation in MRC-5 cells (49.8 ± 9.4%, *P* < 0.05), and at 48 h (27.3 ± 7.2%, *P* < 0.05) and 72 h (28.5 ± 6.5%, *P* < 0.05) in NHLFs, respectively (Fig. 3a). We confirmed that periostin silencing did not weaken adhesion of MRC-5 cells (3.467 × 10^3^ ± 242.5 for control vs. 2.494 × 10^3^ ± 231.6 for periostin). Moreover, we confirmed downregulation of cell proliferation by periostin silencing by the BrdU assay (Fig. 3b). However, we could not observe enhancement of cell proliferation of MRC-5 cells by adding recombinant periostin protein (Additional file 1: Figure S2).

To exclude the possibility that the decrease of cultured cell numbers by periostin knockdown is due to induced cell death, we next investigated whether periostin silencing causes cell death, using double staining of annexin V and PI. We confirmed that addition of both cycloheximide and TNF-α increased the annexin V^+^ and PI^−^ fraction, suggesting induction of apoptosis (Fig. 3c). In contrast, knockdown of periostin did not change the numbers of either annexin V^+^ or PI^+^ fraction (Fig. 3d), suggesting that decreased periostin in lung fibroblasts does not cause cell death.

Moreover, we next examined the effect of silencing of α_V_β_3_ integrin, the dominat periostin receptor on lung fibroblasts [15, 27] on the proliferation of MRC-5 cells. Knockdown of integrin α_V_ as much as periostin significantly, and knockdown of integrin β_3_ to a lesser extent, decreased cell proliferation at 72 h (Fig. 3e). Although α_V_β_3_ integrin may play an important role in cell proliferation by binding some ligands other periostin, these results strongly support that the interaction between periostin and integrin would be important for cell proliferation of lung fibroblasts.

### The effect of periostin on cell cycle in lung fibroblasts

**Fig. 4:**
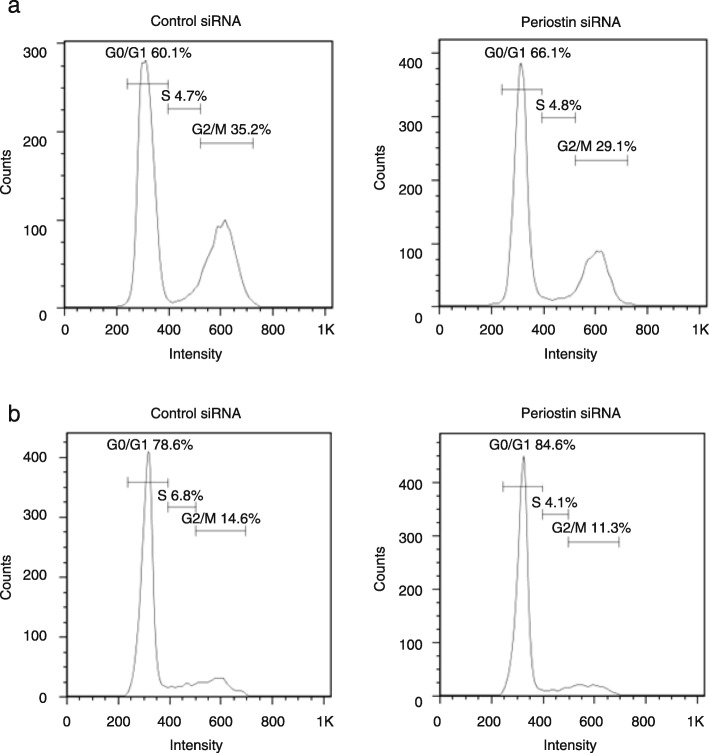
Effect of periostin on the distribution of the cell cycle in lung fibroblasts. After MRC-5 cells and NHLFs were serum-deprived for 24 h, the cells were treated with 10 nM control or periostin siRNA for 48 h. Distribution of the cell cycle of MRC-5 cells (**a**) and NHLFs (**b**) as estimated by flow cytometry is depicted. We performed the same experiments for three times and show the representative data

Given that periostin silencing affects expression of cell-cycle–promoting genes and slows cell proliferation in lung fibroblasts, we examined whether periostin silencing affects distribution of cell cycle in lung fibroblasts. We confirmed that starvation of MRC-5 cells increased the distributions in the G0/G1 phase and decreased those of the G2/M phase (Additional file 1: Figure S3). Periostin silencing increased the distributions in the G0/G1 phase compared to control cells (66.1% vs. 60.1% for MRC-5 cells, 84.6% vs. 78.6% for NHLFs, Fig. 4), whereas the distributions in the G2/M phase were decreased (29.1% vs. 35.2% for MRC-5 cells, 11.4% vs. 14.6% for NHLFs) in both MRC-5 cells and NHLF. The distribution of the S phase either was not changed or decreased slightly (4.7% vs. 4.8% for MRC-5 cells, 6.8% vs. 4.1% for NHLFs). These results suggest that periostin silencing affects the distribution of cell cycle, particularly at the G1/S checkpoint, and drives the cells into G1 arrest, slowing cell proliferation.

### Effect of periostin silencing on cell proliferation in lung fibroblasts derived from IPF patients

**Fig. 5:**
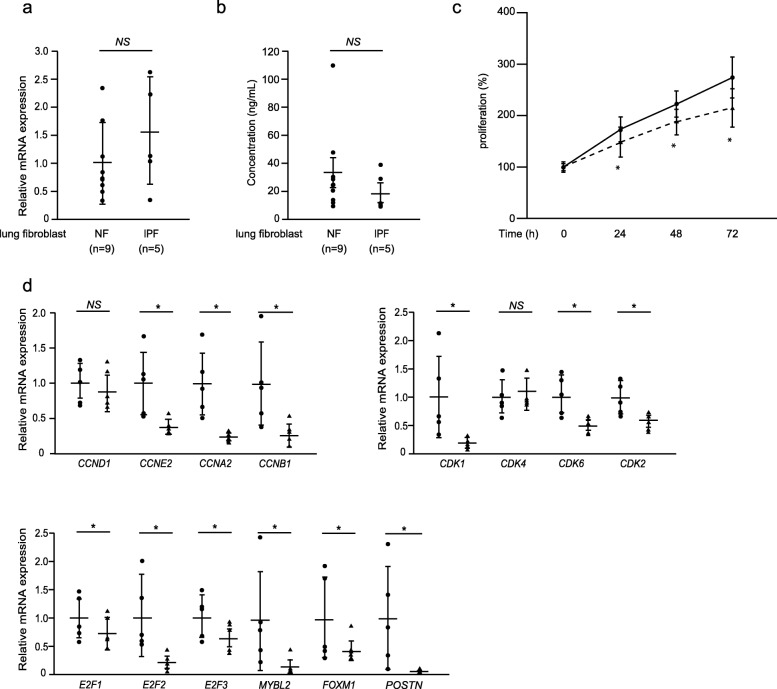
Effect of periostin silencing on proliferation of IPF lung fibroblasts. **a**, **b** Expression of periostin RNA estimated by qRT-PCR or periostin protein estimated by the ELISA assay. The values were adjusted by *GAPDH* expression, and the folds for the values are shown for qRT-PCR in panel **a**. The complete results of nine clones of normal lung fibroblasts and five clones of IPF lung fibroblasts are depicted. Values are mean ± SD of three independent experiments. *P < 0.05. **c** The growth curves of IPF lung fibroblasts. The cells with (dashed line) or without (solid line) knockdown of periostin were plated at a density of 1.0 × 10^4^ cells/well in 96-well plates. The cell numbers were evaluated at the indicated times. Values are mean ± SD of three independent experiments. *P < 0.05. **d** IPF lung fibroblasts were treated with 10 nM control (closed circle) or periostin (closed triangle) siRNA for 48 h. qRT-PCR for the indicated important cell cycle progression genes in periostin-silenced lung fibroblasts. The values were adjusted by *GAPDH* expression, and the folds for the values are shown. Values are mean ± SD of three independent experiments. *P < 0.05, NS: not significant

Given that periostin promotes cell cycle in normal lung fibroblasts, we then looked at whether lung fibroblasts derived from IPF patients also require periostin for maximum proliferation. We first examined periostin expression in five clones of lung fibroblasts derived from IPF patients and in nine clones of those derived from normal donors (Fig. 5a, b). Periostin expression varied among the clones. Although periostin tended to be more highly expressed, but not with statistical significance in IPF lung fibroblasts compared to normal lung fibroblasts at the mRNA level, the amounts of secreted periostin protein were not significantly different between IPF lung fibroblasts and normal lung fibroblasts. We then investigated the effect of periostin silencing on the proliferation of IPF lung fibroblasts. Periostin silencing inhibited proliferation at 24 h (11.8 ± 13.0%, *P* < 0.05), at 48 h (14.8 ± 9.3%, *P* < 0.05), and at 72 h (21.8 ± 7.2%, *P* < 0.05, Fig. 5c). Regardless of the levels of periostin expression, most cell-cycle–related genes behaved the same way as MRC-5 cells: expression of cyclins E2, A2, and B1 in the cyclin family; CDKs 1, 2, and 6 in the CDK family; E2F2 in the E2F family; and B-MYB and FOXM1 was downregulated by periostin silencing in IPF lung fibroblasts as well as in MRC-5 cells (Fig. 5d). Interestingly, although expression of E2F1 and E2F3 tended to be downregulated but did not reach statistical significance by periostin silencing in MRC-5 cells, it was significantly downregulated in IPF lung fibroblasts. These results demonstrate that lung fibroblasts derived from IPF patients sustain the ability to proliferate in a periostin-dependent manner similar to normal lung fibroblasts.

### The effect of CP4715, an inhibitor of integrin α_V_β_3_, on cell proliferation in lung fibroblasts derived from IPF patients

**Fig. 6:**
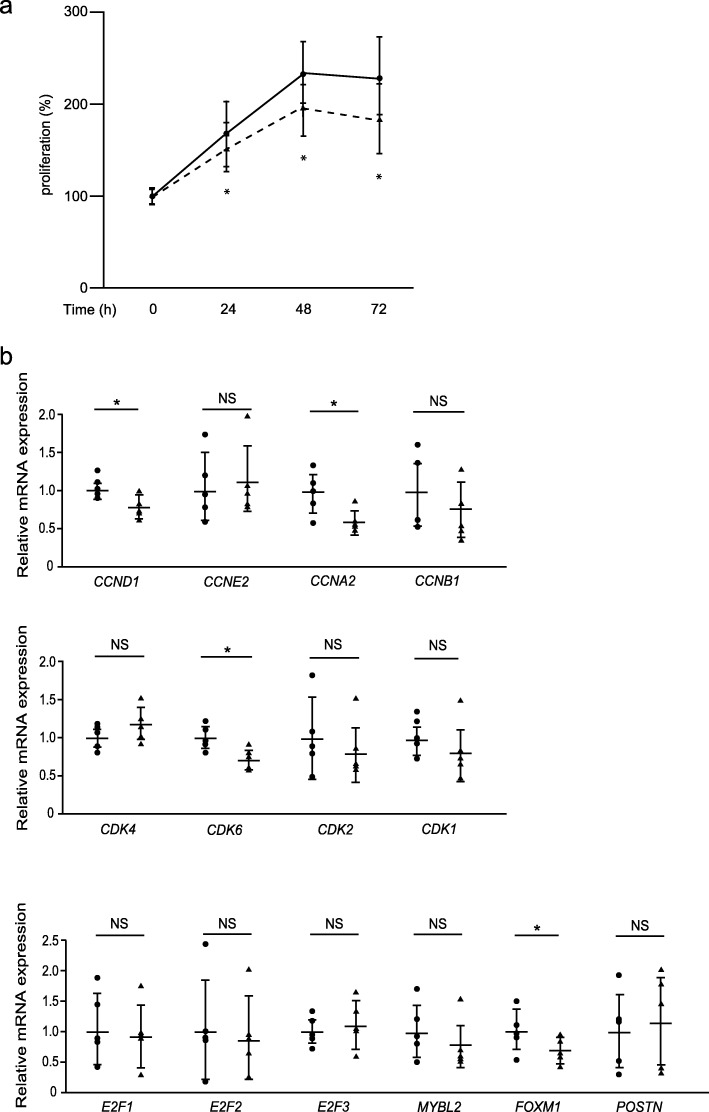
Effect of CP4715 on proliferation of IPF lung fibroblasts. **a** The growth curves of IPF lung fibroblasts. The cells in the absence (solid line) or presence (dashed line) of 1 μM CP4715 dissolved in DMSO were plated at a density of 1.0 × 10^4^ cells/well in 96-well plates. Cell numbers were evaluated at the indicated times. Values are mean ± SD of three independent experiments. *P < 0.05. **b** IPF lung fibroblasts were treated without (closed circle) or with (closed triangle) 1 μM CP4715 dissolved in DMSO for 24 h. qRT-PCR for the indicated important cell cycle progression genes in CP4715-treated lung fibroblasts were performed. The values were adjusted by *GAPDH* expression, and the folds for the values are shown. Values are mean ± SD of three independent experiments. *P < 0.05, **P < 0.01, NS: not significant

We have recently found that CP4715, a potent inhibitor against integrin α_V_β_3_, blocks TGF-β signaling, followed by reduced BLM-induced pulmonary fibrosis in mice, suggesting that CP4715 has the potential to be developed as a therapeutic agent for IPF [15]. Therefore, we investigated whether CP4715 can block proliferation of lung fibroblasts derived from IPF patients. CP4715 inhibited proliferation of IPF lung fibroblasts after 24 h (14.6 ± 8.9%, *P* < 0.05), after 48 h (19.3 ± 10.1%, *P* < 0.05), and after 72 h (29.1 ± 12.3%, *P* < 0.05) at the same levels as periostin silencing (Fig. 6a). However, the ability to downregulate cell-cycle–related genes was weaker than periostin silencing (Fig. 6b); CP4715 downregulated expression of cyclin A2 in the cyclin family, CDK 6 in the CDK family, and FOXM1 as well as periostin silencing did. However, expression of cyclins E2 and B1; CDKs 1 and 2; all of E2F1, E2F2, and E2F3; and B-MYB was not changed or tended to be downregulated without statistical significance by CP4715 treatment, in contrast to periostin silencing. These results demonstrate that CP4715 can inhibit proliferation of IPF lung fibroblasts as well as periostin silencing but has a weaker ability to downregulate cell-cycle–related genes.

## Discussion

Periostin is a matricellular protein that exerts various effects on cells by binding to several integrins on the cell surface [6]. The ability of periostin to promote cell growth in cancer cells has been well studied; either exposure of periostin, transfection of the periostin gene, or co-existence of periostin-producing cells can enhance proliferation of cancer cells [28–33]. This may be an underlying mechanism explaining why, in cancer, high periostin levels reflect aggressive tumor behavior, advanced stage, and poor prognosis [34]. Activation of the Erk pathways and the cross-talk with EGF signals have both been proposed as the underlying mechanism of how periostin accelerates proliferation of cancer cells [31–33]. Moreover, it has been reported that periostin induces cell cycle reentry in cardiomyocytes, followed by improving ventricular remodeling and cardiac function after myocardial infarction [35], although these effects are still controversial [36]. Our present study shows that periostin is required for maximal proliferation of normal lung fibroblasts and, moreover, that IPF lung fibroblasts retain this activity. We found that neither exposure to periostin nor overexpression of periostin enhances proliferation or expression of cell cycle-related genes in lung fibroblasts (Additional file 1: Figures S1 and S2). These results may suggest that the cell cycle in lung fibroblasts is strictly regulated compared with cancer cells [37], and that excess amounts of periostin do not add proliferative effects on lung fibroblasts in vitro. It has been reported that the negatively regulatory mechanism of cell cycle is impaired in IPF patients [38]. In such a situation, stimulation by periostin may enhance cell proliferation of lung fibroblasts. The finding that lung structure is normally maintained in periostin-deficient mice points to a dispensable role for periostin on proliferation of lung fibroblasts at steady state [14]. However, given the aggressive status of proliferation for lung fibroblasts such as IPF, expression level of periostin may make a difference in expansion of fibroblasts.

It is widely accepted that the mitogen and integrin signals via the PI3K/Akt and Erk pathways are important for the transition of the cell cycle from the G1 to the S phase, the first checkpoint of the cell cycle [39]. Integrin signals are unique among integrin members [39, 40] and periostin is a unique ligand for α_V_β_3_ because periostin does not have an RGD sequence like other ligands such as vitronectin, osteopontin, and fibronectin. Nevertheless, the cell cycle analysis in the present study shows that periostin is important for the G1/S transition of the cell cycle as well as other integrin ligands (Fig. 4). In the G1 phase, the cyclin D1/CDK4 complex phosphorylates Rb protein, followed by dissociation of E2F from Rb [36]. E2F increases transcription of cyclin E followed by formation of the cyclin E/CDK2 complex, which furthers Rb phosphorylation. Our present study shows that periostin is important for the expression of cyclin E2, CDK2, and E2F members, but not of cyclin D1 or CDK4. These results suggest that periostin promotes G1/S transition by enhancing production of the cyclin E/CDK2 complex via E2F members, rather than via the cyclin D/CDK4 complex. Although periostin is important for expression of the G2/M phase–related molecules―cyclin B, CDK1, B-MYB, and FOXM1―periostin silencing is unlikely to cause obvious impairment of the G2/M checkpoint.

It is appreciated that fibroblasts taken from IPF patients and cultured in vitro still retain the characteristics of the fibroblasts in vivo in IPF patients. IPF fibroblasts display enhanced proliferation on polymerized collagen matrices [41]. Moreover, profiles of expressed genes differ between IPF patients and normal donors [3–5] . These differences include several signature molecules of IPF such as IGFBP-3 and lysyl oxidase [3]. Lee et al. have reported that periostin expression is enhanced in fibroblasts derived from IPF patients, although expression levels of periostin vary among the clones [5]. We observed that some clones of IPF lung fibroblasts show high expression of periostin compared to normal lung fibroblasts, whereas there was no statistical significance because of the heterogeneity of IPF lung fibroblasts (Fig. 5). The concept of the heterogeneity of IPF lung fibroblasts is consistent with our previous finding that high expression of periostin is relatively limited to the fibroblastic foci, which are not broadly observed in the lungs of IPF patients [7]. In spite of the heterogeneity of periostin expression in IPF lung fibroblasts, all IPF lung fibroblasts retain the effects of periostin silencing on cell growth and expression of cell-cycle–related molecules (Fig. 5). These results suggest that neither programming nor the extracellular milieu in IPF affect the signal pathway of periostin for proliferation of lung fibroblasts.

Sadly, the median survival for IPF patients is only 3 to 5 years. Thus far, only two drugs, pirfenidone and nintedanib, have been approved by by FDA to treat IPF, and the efficacy of these drugs is limited. There is still an unmet need to develop a novel and effective therapeutic drug to treat IPF. Given that periostin is a key molecule in the pathogenesis of pulmonary fibrosis, it is a promising therapeutic target for IPF. Building on this concept, we have recently found that cross-talk between TGF-β and periostin is important for the generation of pulmonary fibrosis and that CP4715, a potent inhibitor of integrin α_V_β_3_, improves pulmonary fibrosis in mice by inhibiting TGF-β signaling [15]. Our present study shows that CP4715 has a potent ability to slow proliferation of IPF fibroblasts, as does periostin silencing, although CP4715 has weaker abilities to downregulate cell-cycle–related genes than periostin silencing (Fig. 6). These results give us a basis for applying inhibitors of the periostin/integrin α_V_β_3_ interaction to IPF patients.

## Conclusions

It is still uncertain how the fibroblasts in the patients with idiopathic pulmonary fibrosis (IPF) acquire the ability to progressively proliferate. Periostin is a matricellular protein playing a critical role in the pathogenesis of pulmonary fibrosis. In this study, we found that periostin plays a critical role in the proliferation of lung fibroblasts and that inhibition of the periostin/integrin α_V_β_3_ (a periostin receptor) interaction can be useful for the treatment of IPF patients.

## Supplementary information

**Additional file 1: Figure S1.** Effect of periostin overexpression on expression of cell cycle-related genes in lung fibroblasts. MRC-5 cells were transiently transfected with 0.1 μg of the mock plasmid (open bar) or the expression plasmid encoding periostin (closed bar). qRT-PCR for the indicated important cell cycle progression genes were performed in periostin-overexpressed MRC-5 cells after 48 h. The values were adjusted by *GAPDH* expression, and the fold changes are shown. Values are mean ± SD of three independent experiments. NS: not significant. **Figure S2.** Effect of adding recombinant periostin protein on proliferation of lung fibroblasts. The growth curves of MRC-5 cells. (a) The normal cells in the absence (solid line) or presence (dashed line) of recombinant periostin protein (10 μg/mL) were plated at a density of 1.0 ×10^4^ cells/well in 96-well plates. (b) The control cells (black solid line) or periostin knockdown cells with (gray solid line) or without (black dashed line) recombinant periostin protein (10 μg/mL) were plated at a density of 1.0 ×10^4^ cells/well in 96-well plates. Cell numbers were evaluated at the indicated times. Values are mean ± SD of three independent experiments. *P < 0.05, NS: not significant. **Figure S3.** Effect of serum starvation on the distribution of the cell cycle in lung fibroblasts. MRC-5 cells were cultured in the medium with (left panel) or without (right panel) serum for 48 h. Distribution of the cell cycle of MRC-5 cells as estimated by flow cytometry is depicted. We performed the same experiments for three times and show the representative data.

**Additional file 2: Table S1.** Primer sequences used in qRT-PCR.

**Additional file 3: Table S2.** The profile of genes downregulated to less than one third or upregulated by more than three-fold by knockdown of periostin using DNA microarrays.

## Data Availability

The datasets used and/or analyzed during the current study available from the corresponding author on reasonable request.

## Abbreviations

Ab: Antibody
BLM: Bleomycin
CDC: Cell division cycle
CDK: Cyclin-dependent kinase
ECM: Extracellular matrix
IPF: Idiopathic pulmonary fibrosis
NHLF: Normal human lung fibroblast
PI: Propidium iodide
qRT-PCR: Quantitative reverse transcription PCR
