# Supplementary figures and images for: Periostin plays a critical role in the cell cycle in lung fibroblasts
Source: Respir Res. 2020 Jan 30;21:38. doi: 10.1186/s12931-020-1299-0 (PMC6993476; doi:10.1186/s12931-020-1299-0)

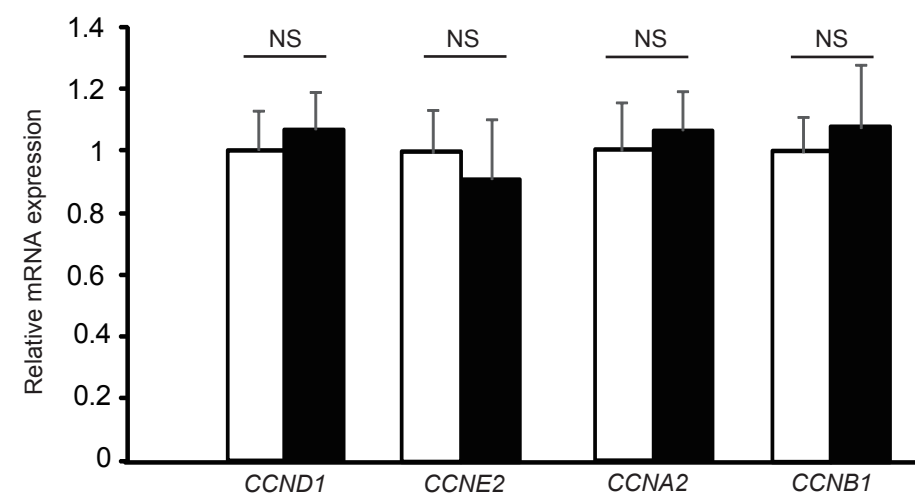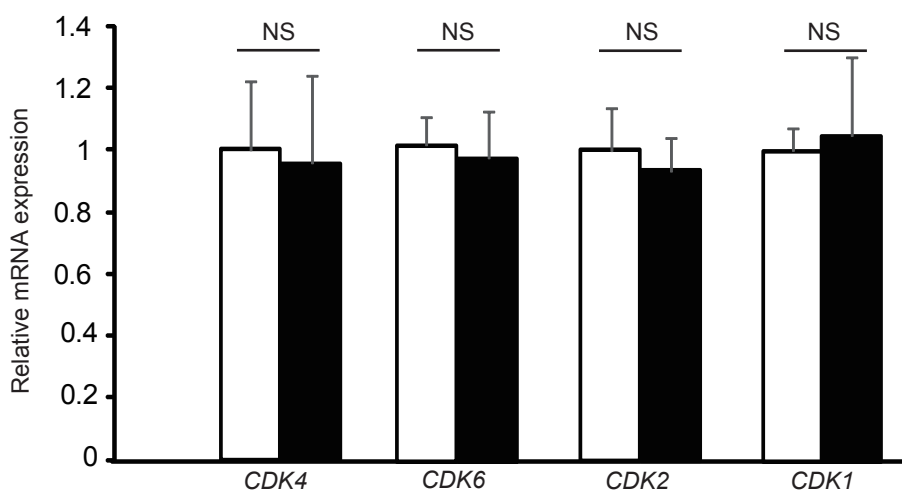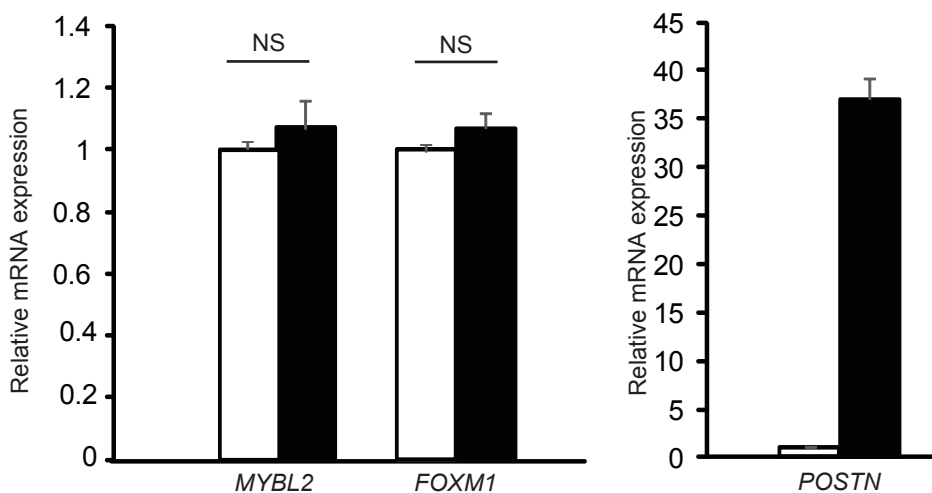

a

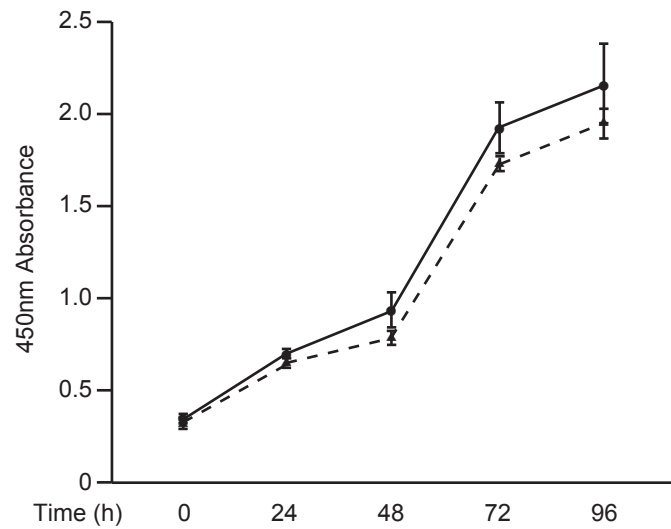

b

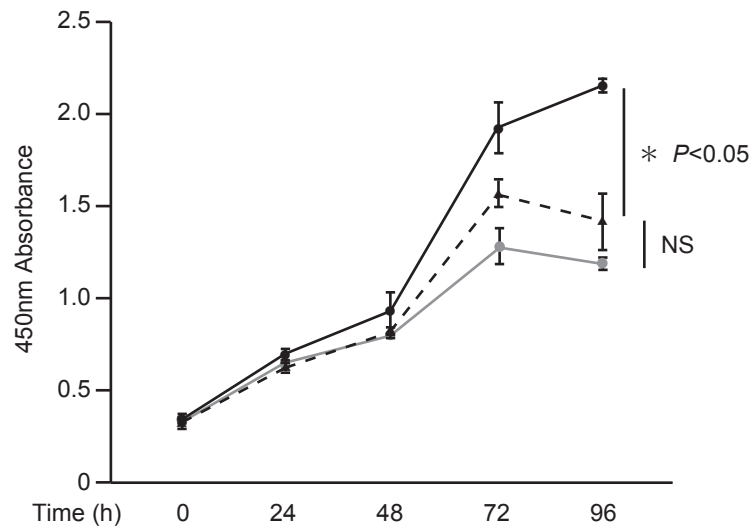

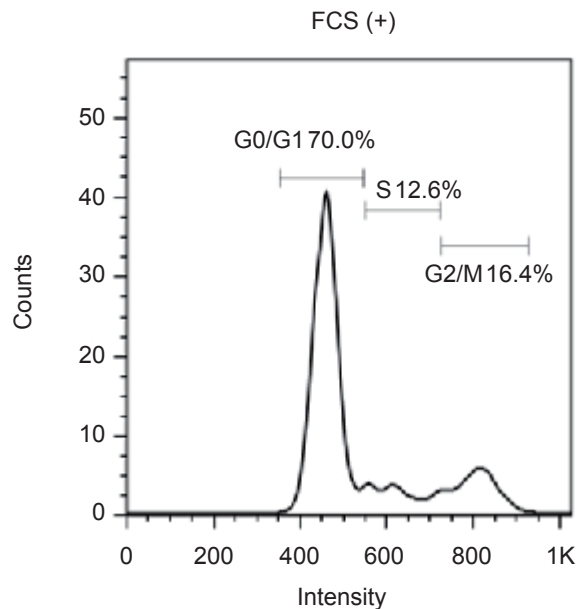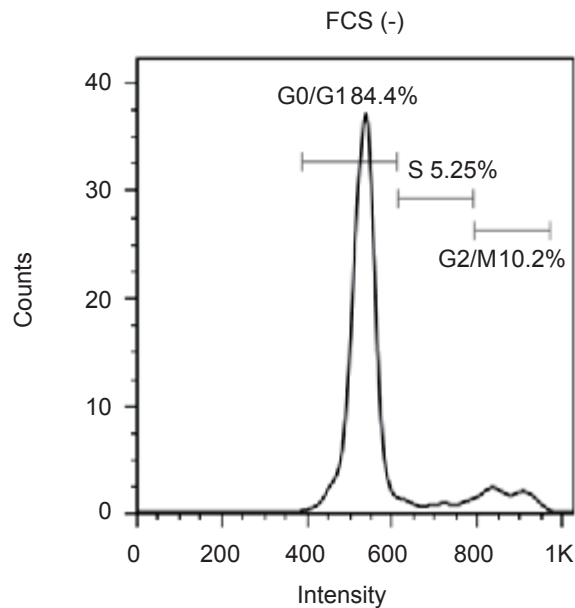

Supplement: Supplementary file 1 — Additional file 1: Figure S1. Effect of periostin overexpression on expression of cell cycle-related genes in lung fibroblasts. MRC-5 cells were transiently transfected with 0.1 μg of the mock plasmid (open bar) or the expression plasmid encoding periostin (closed bar). qRT-PCR for the indicated important cell cycle progression genes were performed in periostin-overexpressed MRC-5 cells after 48 h. The values were adjusted by GAPDH expression, and the fold changes are shown. Values are mean ± SD of three independent experiments. NS: not significant. Figure S2. Effect of adding recombinant periostin protein on proliferation of lung fibroblasts. The growth curves of MRC-5 cells. (a) The normal cells in the absence (solid line) or presence (dashed line) of recombinant periostin protein (10 μg/mL) were plated at a density of 1.0 ×10^4^ cells/well in 96-well plates. (b) The control cells (black solid line) or periostin knockdown cells with (gray solid line) or without (black dashed line) recombinant periostin protein (10 μg/mL) were plated at a density of 1.0 ×10^4^ cells/well in 96-well plates. Cell numbers were evaluated at the indicated times. Values are mean ± SD of three independent experiments. *P < 0.05, NS: not significant. Figure S3. Effect of serum starvation on the distribution of the cell cycle in lung fibroblasts. MRC-5 cells were cultured in the medium with (left panel) or without (right panel) serum for 48 h. Distribution of the cell cycle of MRC-5 cells as estimated by flow cytometry is depicted. We performed the same experiments for three times and show the representative data. [file 12931_2020_1299_MOESM1_ESM.pdf]
