# Supplementary material for: Periostin plays a critical role in the cell cycle in lung fibroblasts
Source: Respir Res. 2020 Jan 30;21:38. doi: 10.1186/s12931-020-1299-0 (PMC6993476; doi:10.1186/s12931-020-1299-0)
Supplement: Supplementary file 2 — Additional file 2: Table S1. Primer sequences used in qRT-PCR. [file 12931_2020_1299_MOESM2_ESM.pdf]

Table S1 Primer sequences used in qRT-PCR

| Gene | Primer sequence |
|---|---|
| GAPDH | Forward Primer: 5’ – TGCACCACCAACTGCTTAGC – 3’<br>Reverse Primer: 5’ – GGCATGGACTGTGGTCATGAG – 3’ |
| POSTN | Forward Primer: 5’ – CAGAGAAATCCCTCCATGAAA – 3’<br>Reverse Primer: 5’ – CAGGAGCTCTTTCAAGTCYGC – 3’ |
| CCNA2 | Forward Primer: 5’ – CCTGCAAACTGCAAAGTTGA – 3’<br>Reverse Primer: 5’ – AAAGGCAGCTCCAGCAATAA – 3’ |
| CCNB1 | Forward Primer: 5’ – AAGCCCAATGGAAACATCTG – 3’<br>Reverse Primer: 5’ – GGATCAGCTCCATCTTCTGC – 3’ |
| CCND1 | Forward Primer: 5’ – AACTACCTGGACCGCTTCCT – 3’<br>Reverse Primer: 5’ – CCACTTGAGCTTGTTCACCA – 3’ |
| CCNE2 | Forward Primer: 5’ – CAGGTTTGGAGTGGGACAGT – 3’<br>Reverse Primer: 5’ – CTCCATTGCACACTGGTGAC – 3’ |
| CDK1 | Forward Primer: 5’ –GGTCAAGTGGTAGCCATGAAA – 3’<br>Reverse Primer: 5’ –TCCTGCATAAGCACATCCTG – 3’ |
| CDK2 | Forward Primer: 5’ – CATTCCTCTTCCCCTCATCA – 3’<br>Reverse Primer: 5’ – CAGGGACTCCAAAAGCTCTG – 3’ |
| CDK4 | Forward Primer: 5’ – GAAACTCTGAAGCCGACCAG – 3’<br>Reverse Primer: 5’ – AGGCAGAGATTCGCTTGTGT – 3’ |
| CDK6 | Forward Primer: 5’ – TGCACAGTGTCACGAACAGA – 3’<br>Reverse Primer: 5’ – ACCTCGGAGAAGCTGAAACA – 3’ |
| E2F1 | Forward Primer: 5’ – TGCAGAGCAGATGGTTATGG – 3’<br>Reverse Primer: 5’ – CTCAGGGCACAGGAAAACAT – 3’ |
| E2F2 | Forward Primer: 5’ – CCTTGGAGGCTACTGACAGC – 3’<br>Reverse Primer: 5’ – CCACAGGTAGTCGTCCTGGT – 3’ |
| E2F3 | Forward Primer: 5’ – GGGAAACCTTTCTCCTCTGG – 3’<br>Reverse Primer: 5’ – GGGGAGGCAGTAAGTTCACA – 3’ |
| MYBL2 | Forward Primer: 5’ – CCATGGACCAAAGAGGAAGA – 3’<br>Reverse Primer: 5’ – CTCAGGGTTGAGGTGGTTGT – 3’ |
| FOXM1 | Forward Primer: 5’ – TCTCGGAGGAAACAGCATCT – 3’<br>Reverse Primer: 5’ – CAGAGGAGTCTGCTGGGAAC – 3’ |
